# Susceptibility to echinocandins of *Candida* spp. strains isolated in Italy assessed by European Committee for Antimicrobial Susceptibility Testing and Clinical Laboratory Standards Institute broth microdilution methods

**DOI:** 10.1186/s12866-015-0442-4

**Published:** 2015-05-20

**Authors:** Maria Teresa Montagna, Grazia Lovero, Caterina Coretti, Domenico Martinelli, Osvalda De Giglio, Roberta Iatta, Stella Balbino, Antonio Rosato, Giuseppina Caggiano

**Affiliations:** Department of Biomedical Science and Human Oncology, Hygiene Section, University of Bari “Aldo Moro”, Piazza Giulio Cesare 11, Bari, Italy; Department of Medical and Surgical Sciences, Hygiene Section, University of Foggia, Via Gramsci 89-91, 71100 Foggia, Italy; Department of Veterinary Medicine, University of Bari Aldo Moro, Str. Prov. per Casamassima Km 3, Valenzano, Bari, Italy; Department of Pharmacy-Drug Sciences, University of Bari Aldo Moro, Campus-Via Orabona, 4, Bari, Italy

**Keywords:** *Candida* spp., EUCAST, CLSI, Anidulafungin, Caspofungin, Micafungin, Antifungal

## Abstract

**Background:**

The echinocandins are recommended as first-line therapy for *Candida* species infections, but drug resistance, especially among *Candida glabrata*, is becoming more frequent. We investigated the antifungal susceptibility of anidulafungin, caspofungin, and micafungin against 584 isolates of *Candida* spp. (bloodstream, other sterile sites) collected from patients admitted to an Italian university hospital between 2000 and 2013. The susceptibility was evaluated using the broth microdilution method according to both the European Committee for Antimicrobial Susceptibility Testing (EUCAST EDef 7.2) and the Clinical Laboratory Standards Institute (CLSI M27-A3). The echinocandin susceptibilities were assessed on the basis of the species-specific clinical breakpoints proposed by the EUCAST version 6.1 and CLSI M27-S4 documents. The two methods were comparable by assessing essential agreement (EA), categorical agreement (CA), and Spearman’s correlation analysis (rho, r).

**Results:**

The modal minimum inhibitory concentrations (MICs; μg ⋅ mL ^−1^) values by both methods (EUCAST/CLSI) for anidulafungin, caspofungin, and micafungin for each species were, respectively, as follows: *C. albicans*, 0.03/0.12, 0.016/0.5, and 0.016/0.008; *C. parapsilosis* complex, 2/1, 2/2, and 2/1; *C. tropicalis*, 0.06/0.12, 0.06/0.12, and 0.06/0.12; *C. glabrata* complex, 0.03/0.25, 0.06/0.12, and 0.03/0.06; *C. guilliermondii*, 2/1, 2/2, and 2/2; and *C. krusei*, 0.06/0.12, 0.12/0.5, and 0.06/0.12. The overall resistance rates for EUCAST/CLSI were as follows: anidulafungin, 2.5/0.9 %; caspofungin, breakpoint not available/3.8 %; micafungin, 2.7/1.5 %.

*Candida glabrata* complex was the least susceptible to all three echinocandins, and the percentages of resistant isolates by EUCAST/CLSI were as follows: anidulafungin, 13.5/2.7 %; caspofungin, breakpoint not available/16.2 %; micafungin, 18.9/13.5 %. The overall EA was 93 % for micafungin, 92 % for anidulafungin, and 90 % for caspofungin. The CA was >90 % for all organism-drug combinations with the exception of *C. glabrata* and anidulafungin (89 %). Spearman’s rho for EUCAST/CLSI was 0.89 (*p* < 0.001) for caspofungin, 0.85 (*p* < 0.001) for anidulafungin, and 0.83 for micafungin (*p* < 0.001).

**Conclusions:**

Independent of the procedure applied, no alarming resistance to the tested agents was found, although a reduced susceptibility was detected for *C. glabrata* complex*.* The EUCAST and CLSI methods produce similar MICs, indicating that using one method or the other should not result in susceptibilities different enough to affect treatment decisions.

## Background

The echinocandins (anidulafungin [AND], caspofungin [CSP], and micafungin [MCF]) are lipopeptides that inhibit glucan synthase, which is responsible for the biosynthesis of β-1,3-D-glucan, a major structural component of fungal cell walls. These drugs demonstrate fungicidal activity against most species of *Candida* and are effective against azole-resistant yeasts and *Candida*-forming biofilms [1–5]. The Infectious Diseases Society of America [6] and the European Society of Clinical Microbiology and Infectious Diseases [7] guidelines for the management of *Candida* infection recommend echinocandins for first-line therapy [8].

Two reference methods analyze the susceptibility of yeasts to echinocandins: the broth microdilution (BMD) method designed by the Clinical Laboratory Standards Institute (CLSI) [9, 10] and the method proposed by the European Committee for Antimicrobial Susceptibility Testing (EUCAST) [11–13]. These methods have some aspects in common: the use of BMD, the use of RPMI 1640 broth as a basal medium, a 24-h incubation period, and a prominent inhibition (50 % relative to the growth control) minimum inhibitory concentration (MIC) endpoint criterion. The main differences are as follows: inoculum density (CLSI, 0.5 × 10^3^ to 2.5 × 10^3^ cells ⋅ mL^−1^; EUCAST, 0.5 × 10^5^ to 2.5 × 10^5^ cells ⋅ mL^−1^); glucose content of the medium (CLSI, 0.2 %; EUCAST, 2.0 %); microdilution wells (CLSI, round-bottom wells; EUCAST, flat-bottom wells); and endpoint reading (CLSI, visual reading; EUCAST, spectrophotometric reading). Moreover, EUCAST breakpoints for AND and MCF are lower than the CLSI breakpoints; additionally, EUCAST has not proposed clinical breakpoints for CSP.

Until now, in Italy, there is not an extensive study concerning the *in vitro* susceptibility of echinocandins against *Candida* isolates using the EUCAST method [14]. The aims of this study are: i) to determine the susceptibilities of *Candida* spp. to AND, CSP, and MCF using both the EUCAST and CLSI methods; and ii) to compare the performance of both methods by assessing the essential agreement (EA) and categorical agreement (CA) levels.

## Results and discussion

In each experiment, the MIC values of the quality control strains fell within the established ranges published for both methods [9, 13]. The modal MIC (μg ⋅ mL ^− 1^; 15 repetitions) values by EUCAST/CLSI for AND, CSP, and MCF for each control strain were, respectively, as follows: *C. krusei* ATCC 6258, 0.06/0.12, 0.5/0.5, and 0.12/0.12; *C. parapsilosis* ATCC 22019, 1/1, 0.5/1, and 0.5/0.5. Table 1 summarizes the *in vitro* susceptibility values of 584 clinical isolates of *Candida* species to echinocandins as determined by the CLSI and EUCAST BMD methods, as well as EA, and CA between two methods for each echinocandin and *Candida* species. Generally, the MIC values for all three echinocandins were low and below the susceptibility breakpoint, regardless of the method used. As shown by other authors [15], the MIC values for AND (geometric mean MIC EUCAST/CLSI, 0.16/0.22 μg ⋅ mL^−1^) and MCF (geometric mean MIC EUCAST/CLSI, 0.13/0.14 μg ⋅ mL^−1^) were lower those for CSP (geometric mean MIC EUCAST/CLSI, 0.29/0.33 μg ⋅ mL^−1^) by both assay, suggesting that they have superior *in vitro* potency. When the species-specific clinical breakpoints were applied, the overall resistance rates for EUCAST/CLSI were as follows: AND, 2.5/0.9 %; CSP, breakpoint not available/3.8 %; MCF, 2.7/1.5 %. These data are consistent with those reported previously [14, 16–18] and document the excellent potency and spectrum of echinocandins against most *Candida* spp. Of the 22 CSP resistant isolates by CLSI, five were MCF resistant, three were AND resistant, and one was resistant both AND and MCF; this discrepant susceptibility pattern is according to other studies [17, 19]. Given the mechanism of action that is shared among the echinocandins, it is biologically unlikely that such large percentages of isolates are non susceptible to CSP but remain susceptible to AND and MCF. A potential explanation for this finding may be the technical issues associated with the *in vitro* testing of CSP rather than a true difference in antifungal activity [20]. For this reason, neither EUCAST nor CLSI procedures recommend the use of CSP for antifungal susceptibility testing, while AND and MCF as markers for CSP susceptibility [11, 21].

**Table 1:** Agreement between the results of the European Committee for Antimicrobial Susceptibility Testing and Clinical Laboratory Standards Institute broth microdilution methods for anidulafungin, caspofungin, and micafungin

Isolates (No.)	Antifungal drug	BMD method	MIC (μg mL^−1^)				No. (%) S	No. (%) I	No. (%) R	EA (%)	CA (%)
			Range	Mode	GM	MIC_90_					
*C. albicans* (251)	Anidulafungin	CLSI	≤0.008–2	0.12	0.03	0.12	246(98)	2(0.8)	3(1.2)	90	99
		EUCAST	≤0.008–2	0.03	0.02	0.03	247(98.4)	NA	4(1.6)		
	Micafungin	CLSI	≤0.008–1	0.008	0.01	0.12	241(96)	7(2.8)	3(1.2)	92	98
		EUCAST	≤0.008–0.5	0.016	0.01	0.016	244(97.2)	NA	7(2.8)		
	Caspofungin	CLSI	≤0.008–4	0.5	0.17	0.5	180(71.7)	63(25.1)	8(3.2)	87	NA
		EUCAST	≤0.008–2	0.016	0.13	0.5	−	−	−		
*C. parapsilosis complex* (224)	Anidulafungin	CLSI	0.03–2	1	1.14	2	224(100)	0	0	96	100
		EUCAST	0.06–2	2	1.78	2	0	224(100)	0		
	Micafungin	CLSI	0.016–2	1	0.96	2	224(100)	0	0	95	100
		EUCAST	0.016–2	2	1.62	2	0	224(100)	0		
	Caspofungin	CLSI	0.25–8	2	1.62	2	217(96.9)	6(2.7)	1(0.4)	98	NA
		EUCAST	0.06–8	2	1.82	2	−	−	−		
*C. tropicalis* (46)	Anidulafungin	CLSI	≤0.008–1	0.12	0.09	0.25	45(97.8)	0	1(2.2)	91	93
		EUCAST	≤0.008–0.25	0.06	0.05	0.06	42(91.3)	NA	4(8.7)		
	Micafungin	CLSI	≤0.008–1	0.12	0.07	0.5	37(80.4)	(17.4)	1(2.2)	91	NA
		EUCAST	0.016–2	0.06	0.11	0.5	−	−	−		
	Caspofungin	CLSI	≤0.008–2	0.12	0.12	1	34(74)	6(13)	6(13)	89	NA
		EUCAST	≤0.008–2	0.06	0.11	1	−	−	−		
*C. glabrata* complex (37)	Anidulafungin	CLSI	≤0.008–4	0.25	0.13	0.25	24(64.9)	12(32.4)	1(2.7)	72	89
		EUCAST	≤0.008–2	0.03	0.06	0.12	32(86.5)	NA	5(13.5)		
	Micafungin	CLSI	≤0.008–1	0.06	0.06	0.12	28(75.7)	4(10.8)	5(13.5)	83	95
		EUCAST	≤0.008–2	0.03	0.05	0.12	30(81.1)	NA	7(18.9)		
	Caspofungin	CLSI	≤0.008–4	0.12	0.18	1	21(56.8)	10(27)	6(16.2)	80	NA
		EUCAST	≤0.008–2	0.06	0.17	2	−	−	−		
*C. guilliermondii* (15)	Anidulafungin	CLSI	1–2	1	1.45	2	14(93.3)	1(6.7)	0	100	NA
		EUCAST	1–2	2	1.91	2	−	−	−		
	Micafungin	CLSI	0.5–2	2	1.45	2	15(100)	0	0	100	NA
		EUCAST	0.5–2	2	1.52	2	−	−	−		
	Caspofungin	CLSI	0.12–4	2	1.74	2	14(93.3)	1(6.7)	0	93	NA
		EUCAST	1–2	2	1.74	2	−	−	−		
*C. krusei* (11)	Anidulafungin	CLSI	0.12–0.5	0.12	0.14	0.12	10(90.9)	1(9)	0	90	91
		EUCAST	0.06–0.12	0.06	0.06	0.06	10(90.9)	NA	1(9.1)		
	Micafungin	CLSI	0.06–0.5	0.12	0.13	0.12	11(100)	0	0	100	NA
		EUCAST	0.03–0.5	0.06	0.08	0.25	−	−	−		
	Caspofungin	CLSI	0.12–2	0.5	0.44	2	6(54.5)	4(36.4)	1(9.1)	90	NA
		EUCAST	0.06–2	0.12	0.23	1	−	−	−		

“−” denotes that no breakpoints have yet been established; BMD, broth microdilution; CLSI, Clinical and Laboratory Standards Institute; EUCAST, European Committee on Antimicrobial Susceptibility Testing; MIC, minimum inhibitory concentration; GM, geometric mean; EA, essential agreement; CA, categorial agreement; NA, Not applicable; S, susceptible; I, intermediate; R, resistant

The trend in the rate of resistance to echinocandins was analyzed: no significant trend was observed for each drug over the studied 14-year period, by both methods.

The MIC values were the highest for *C. parapsilosis* complex and *C. guilliermondii* by the two methods. A natural polymorphism occurring in the hot spot, one region of *FKS*1 has been suggested to be responsible for the reduced echinocandin susceptibilities of these species [22]. Unlike yeasts with acquired *FKS* mutations, these strains respond well to standard therapy presumably because the polymorphism only weakly affects the sensitivity of glucan synthase for drug [5]. Moreover, the good clinical response of *C. parapsilosis* may be due to its less virulent, reduced capacity to invade the deep tissue, and the high probability of therapeutic success if central venous catheter is removed [15].

In our study, the resistance to AND (EUCAST/CLSI, 13.5/2.7 %), CSP (EUCAST/CLSI, breakpoint not available/16.2 %), and MCF (EUCAST/CLSI, 18.9/13.5 %) was most prominent among *C. glabrata* complex isolates. Similarly, the SENTRY Antimicrobial Surveillance Program reported echinocandin resistance of 8.0–9.3 % among 1669 *C. glabrata* from blood stream infections [23]; moreover, in a 10-year survey at the Duke university hospital echinocandin resistance rate increased from 4.9 to 12.3 % in 2001–2010 [24]. Prolonged therapy with these drugs has been suggested to be a potential cause for decreased susceptibility to echinocandins among isolates of *C. glabrata* [22–27].

A decrease in the activity of AND (EUCAST/CLSI, 8.7/2.2 %), MCF (EUCAST/CLSI, breakpoint not available/2.2 %), and CSP (EUCAST/CLSI, breakpoint not available/13 %) was also observed among isolates of *C. tropicalis*.

For most isolates, the echinocandin MICs obtained by the EUCAST method tended to be one twofold dilution lower than those obtained by the CLSI method. The reason for this result could be related to known differences between the two methods: the higher carbohydrate content in the RPMI and higher inoculum could be responsible for the lowering of the EUCAST results [28].

The overall EA was very high: 93 % for MCF, 92 % for AND, and 90 % for CSP. The Spearman’s correlation analysis shows a significant positive correlation between EUCAST and CLSI MICs (*r* = 0.85, *p* < 0.001 for AND; *r* = 0.89, *p* < 0.001 for CSP; *r* = 0.8, *p* < 0.001 for MCF). This finding was consistent with the results of previous global multicenter studies by Pfaller et al. [29, 30] and confirms the high level of EA between the reference procedures. The rates of EA were also high when results were analyzed per species, and the worst EA (72 %) was for *C. glabrata* complex tested against AND. A good CA was also observed for all organism-drug combinations, ranging from 89 to 100 %. The lowest CA was for *C. glabrata* complex isolates to AND, where five (13.5 %) isolates were resistant when applying EUCAST breakpoints as opposed to one (2.7 %) when applying the CLSI breakpoints. The meaning of this *in vitro* finding is not clear and needs to be clarified in more detail. The best CA was for *C. parapsilosis* complex to AND and MCF.

## Conclusions

Our study had some limitations. First, none of our strains were characterized with respect to echinocandin resistance mechanisms. Second, the lack of EUCAST species-specific breakpoints for CSP precludes a more standard comparison for the assessment of the CA. Third, this study was an observational laboratory based survey, therefore no data was available as to the type and duration of antifungal therapy. Nevertheless, to the best of our knowledge, this is the first study on this topic to be carried out in Italy. Our data show that the EUCAST and CLSI echinocandin MIC values for *Candida* isolates from patients in Italy are in accordance with the worldwide epidemiology of *Candida* strains [14, 16–18]. Likewise, the echinocandins were active *in vitro* against the majority of *Candida* species tested in this study; a reduced susceptibility, as has been noted elsewhere [23, 24], was detected for *C. glabrata* complex and *C. tropicalis*. MIC values obtained by the CLSI and EUCAST methods are comparable for the testing of echinocandins against *Candida* species. Regression analysis confirms the close proximity of the MICs generated by each method. In most cases, the MIC differences between standard procedures are small enough that using one method or the other should not result in susceptibilities that are different enough to affect treatment decisions. The clinical implications regarding the improvement of the susceptibility tests to echinocandins are significant, since accurate data are important in defining differences in clinical efficacy among AND, CSP and MCF, thereby supporting the most appropriate choice of early antifungal treatment towards a better prognosis. Further efforts are needed to harmonize the two standard procedures.

## Methods

### Clinical isolates

Between January 2000 and December 2013, a total of 597 clinical isolates of *Candida* spp. (bloodstream and other sterile sites) were collected from patients admitted to a large Italian university hospital. Of these, the six most common *Candida* species were tested against echinocandins (251 *C. albicans*, 224 *C. parapsilosis*, 46 *C. tropicalis*, 37 *C. glabrata*, 15 *C. guilliermondii*, 11 *C. krusei*), for a total of 584 isolates recovered from the following wards: intensive care unit (*n* = 288), haematology (*n* = 99), internal medicine (*n* = 88), surgery (*n* = 85), and oncology (*n* = 24). For this study, we did not use any additional data or samples other than those obtained through routine laboratory collection. Therefore, neither ethical approval nor patient consent was considered necessary. Registered data were managed in accordance with the Italian data protection laws (privacy law). The isolates were identified using standard procedures (*i.e.*, morphology on cornmeal agar plates, germ-tube production in serum, and ability to grow at 37 °C and 42 °C) and biochemical analysis using the ID32C and VITEK-2 System (Biomérieux, Marcy l’Etoile, France). Each isolate represented a unique strain from a single patient and was frozen at −80 °C until the analysis. Prior to being tested, each isolate was sub-cultured on Sabouraud dextrose agar plates (BioMèrieux) to ensure purity, viability, and optimal growth characteristics.

### Susceptibility testing

AND (Pfizer Pharmaceuticals, Groton, CT, USA), CSP (Merck & Co., Inc., Whitehouse Station, NJ, USA), and MCF (Astellas Pharma, Tokyo, Japan) were obtained as standard powders. A single lot of pure substance for each of the three echinocandins was used. Stock solutions were prepared in dimethyl sulfoxide (Sigma, St. Louis, MO, USA) for each echinocandin, taking into account the potencies of the powders.

CLSI BMD testing was performed according to document M27-A3 [9, 10] using RPMI 1640 medium with 0.2 % glucose (Sigma) and 0.165 M MOPS, an inoculum of 0.5 × 10^3^ to 2.5 × 10^3^ cells ⋅ mL^−1^, and incubation at 35 °C. The MIC values were determined visually after 24 h of incubation as the lowest concentration of drug that caused a significant diminution (≥50 % inhibition) of growth below control levels.

EUCAST BMD testing was performed according to document EDef 7.2 [available on the EUCAST website: http://www.eucast.org] using RPMI 1640 medium supplemented with glucose to a final concentration of 2 %, an inoculum of 0.5 × 10^5^ to 2.5 × 10^5^ cells ⋅ mL ^− 1^, and incubation at 35 °C. MIC values were determined with a spectrophotometer (wavelength of 450 nm; ETI System Fast Reader ELX, Biotek, US) after 24 h of incubation as the lowest concentration of drug that resulted in >50 % inhibition of growth relative to that of the growth control.

*C. krusei* ATCC 6258 and *C. parapsilosis* ATCC 22019 were used as quality control strains in each run according to the CLSI M27-A3 document [9].

### Interpretation and analysis of results

The echinocandin susceptibilities were defined according to the species-specific clinical breakpoints proposed by the EUCAST version 6.1 [31] and CLSI M27-S4 documents [10] (Table 2). Differences in the temporal susceptibility rates were analysed by chi-square test for trend. MIC discrepancies of no more than ± 2-fold dilutions were used to calculate the EA. The CA was defined as the percentage of the discrepancy in the number of resistant isolates based on the existence of interpretative breakpoints [16]. Moreover, to determine the correlation between the methods, Spearman’s rank correlation coefficient (rho, r), and its corresponding p value was performed by plotting EUCAST versus CLSI MICs. The level of significance was set at a p value less than 0.05. Statistical analysis of data was carried out using STATA MP 11.2 for Mac Os X (SPSS Inc., Chicago, IL, USA).

**Table 2:** CLSI (document M27-S4) and EUCAST (version 6.1) antifungal breakpoints for *Candida* species

	MIC (μg/ml) breakpoint for susceptibility/resistance		
Species	Anidulafungin	Caspofungin	Micafungin
*C. albicans*			
CLSI	≤0.25/≥1	≤0.25/≥1	≤0.25/≥1
EUCAST	≤0.03/>0.03	−	≤0.016/>0.016
*C. parapsilosis*			
CLSI	≤2/≥8	≤2/≥8	≤2/≥8
EUCAST	≤0.002/>4	−	≤0.002/>2
*C. glabrata*			
CLSI	≤0.12/≥0.5	≤0.12/≥0.5	≤0.06/≥0.25
EUCAST	≤0.06/>0.06	−	≤0.03/>0.03
*C. tropicalis*			
CLSI	≤0.25/≥1	≤0.25/≥1	≤0.25/≥1
EUCAST	≤0.06/>0.06	−	−
*C. gulliermondii*			
CLSI	≤2/≥8	≤2/≥8	≤2/≥8
EUCAST	−	−	−
*C. krusei*			
CLSI	≤0.25/≥1	≤0.25/≥1	≤0.25/≥1
EUCAST	≤0.06/>0.06	−	−

“−” denotes that no breakpoints have yet been established

## Abbreviations

BMD: Broth microdilution
CLSI: Clinical Laboratory Standards Institute
EUCAST: European Committee for Antimicrobial Susceptibility Testing
AND: Anidulafungin
CSP: Caspofungin
MCF: Micafungin
EA: Essential agreement
CA: Categorical agreement
MIC: Minimum inhibitory concentration
